# Anti-Adipogenic Effects on 3T3-L1 Cells and Zebrafish by Tanshinone IIA

**DOI:** 10.3390/ijms18102065

**Published:** 2017-09-27

**Authors:** Yu-Kyoung Park, Brice Wilfried Obiang-Obounou, Jinho Lee, Tae-Yun Lee, Myung-Ae Bae, Kyu-Seok Hwang, Kyung-Bok Lee, Jong-Soon Choi, Byeong-Churl Jang

**Affiliations:** 1 Department of Molecular Medicine, College of Medicine, Keimyung University, 1095 Dalgubeoldaero, Dalseo-gu, Daegu 42601, Korea; westside1140@hanmail.net; 2 Department of Food Nutrition, College of Natural Sciences, Keimyung University, 1095 Dalgubeoldaero, Dalseo-gu, Daegu 42601, Korea; b_obiang@yahoo.com; 3 Department of Chemistry, College of Natural Sciences, Keimyung University, 1095 Dalgubeoldaero, Dalseo-gu, Daegu 42601, Korea; jinho@gw.kmu.ac.kr; 4 Department of Microbiology, College of Medicine, Yeungnam University, 170 Hyeonchung-ro, Nam-gu, Daegu 42415, Korea; doxr7p@ynu.ac.kr; 5 Bio & Drug Discovery Division, Korea Research Institute of Chemical Technology, 141 Gajeong-ro Yuseong-gu, Daejeon 34114, Korea; mbae@krict.re.kr (M.-A.B.); kshwang@krict.re.kr (K.-S.H.); 6 Biological Disaster Analysis Group, Division of Convergence Biotechnology, Korea Basic Science Institute, 169-148 Gwahak-ro, Yuseong-gu, Daejeon 34133, Korea; kblee@kbsi.re.kr (K.-B.L.); jschoi@kbsi.re.kr (J.-S.C.)

**Keywords:** tanshinone IIA, adipogenesis, 3T3-L1, zebrafish

## Abstract

Tanshinone IIA is a diterpene quinone isolated from the roots of *Salvia miltiorrhiza* bunge that has traditionally been used in China for the treatment of cardiovascular and cerebrovascular disorders. Although there is recent evidence showing that tanshinone IIA has an anti-obesity effect, its underlying mechanism of anti-obesity effect is poorly understood. Here, we investigated the effect of tanshinone IIA on lipid accumulation in 3T3-L1 preadipocytes and zebrafish. Notably, tanshinone IIA at 10 μM concentration greatly reduced lipid accumulation and triglyceride (TG) contents during 3T3-L1 preadipocyte differentiation, suggesting its anti-adipogenic effect. On mechanistic levels, tanshinone IIA reduced the expression levels of CCAAT/enhancer-binding protein-α (C/EBP-α), peroxisome proliferator-activated receptor-γ (PPAR-γ), fatty acid synthase (FAS), and perilipin A but also the phosphorylation levels of signal transducer and activator of transcription-3/5 (STAT-3/5) in differentiating 3T3-L1 cells. In addition, tanshinone IIA strongly inhibited leptin and resistin mRNA expression in differentiating 3T3-L1 cells. Importantly, the tanshinone IIA’s lipid-reducing effect was also seen in zebrafish. In sum, these findings demonstrate that tanshinone IIA has anti-adipogenic effects on 3T3-L1 cells and zebrafish, and its anti-adipogenic effect on 3T3-L1 cells is largely attributable to the reduced expression and/or phosphorylation levels of C/EBP-α, PPAR-γ, FAS, perilipin A, and STAT-3/5.

## 1. Introduction

Obesity is a condition in which excessive body fat accumulation may negatively affect health. The condition is associated with non-communicable diseases, namely hyperlipidemia, type 2 diabetes, and cancer [1]. Genetic/endocrine abnormalities, a low metabolic rate, some medicines, nutritional/environmental factors, and imbalanced energy homeostasis are all factors often mentioned as inducers of obesity [2,3]. Adipose tissue is a loose connective tissue composed mostly of adipocytes [4]. As an energy reservoir and endocrine organ, adipose tissue is known to play an important role in the regulation of energy metabolism for the human body through expression and secretion of an array of adipokines [5,6]. However, excessive expansion of the adipose tissue, largely due to deregulated adipocyte differentiation, is linked to the development of obesity and related disorders. 

Adipocyte differentiation, also called adipogenesis, is the process during which fibroblast-like preadipocytes develop into mature adipocytes [7]. Adipocyte differentiation is controlled by the expressions and activities of the family of CCAAT/enhancer-binding proteins (C/EBPs) and peroxisome proliferator-activated receptors (PPARs) [8,9]. C/EBPα is expressed and involved in the early stage of 3T3-L1 adipocyte differentiation [8,10,11]. PPARγ is known to participate in the regulation of lipid metabolism in adipocytes [12], and its expression and activation in inducing adipogenesis is sufficient as demonstrated by functional and genetic knockdown experiments [13]. In addition, the family of signal transducers and activators of transcription (STATs) is involved in adipogenesis, as evidenced by that the expression and activity (phosphorylation) of STAT-3 and STAT-5 which are markedly elevated during 3T3-L1 adipocyte differentiation [9,14,15]. Moreover, expression and activity (phosphorylation) of STAT-3 is crucial for the early stage of adipogenesis [16] and that of STAT-5 is associated with the adipocyte phenotype [15]. Furthermore, there are recent studies to show that the expression and/or activity of lipogenic enzymes, such as fatty acid synthase (FAS) and lipid droplet (LD) associated proteins like perilipin A, are required for adipocyte differentiation [17,18,19]. 

Tanshinone IIA is a natural diterpene quinone isolated from the roots of *Salvia miltiorrhiza* Bunge that has been used as a traditional Chinese medicine for promotion of blood circulation and relieving vessel stasis [20]. Previous studies have further shown that tanshinone IIA is effective for treating coronary, cerebrovascular, and cardiovascular diseases [21,22,23]. Tanshinone IIA also is known for its anti-oxidant, anti-inflammatory, and anti-cancerous activities [24,25,26,27]. Moreover, of note, there is a previous report advocating that tanshinone IIA has anti-adipogenic and anti-obesity effects on 3T3-L1 cells and high fat diet-induced obese mouse and the effects are mediated through PPAR-γ antagonism [22]. However, at present, the underlying mechanism of tanshinone IIA-mediated anti-adipogenic and anti-obesity effects is still poorly understood. The present study investigates the effect of tanshinone IIA on fat accumulation in 3T3-L1 preadipocytes and zebrafish. Here we demonstrate firstly that tanshinone IIA inhibits lipid accumulation in 3T3-L1 preadipocytes and zebrafish, and its anti-adipogenic effect on 3T3-L1 cells is attributable to the reduced expression and/or phosphorylation levels of C/EBP-α, PPAR-γ, FAS, perilipin A, and STAT-5. 

## 2. Results

### 2.1. Tanshinone IIA Has Strong Anti-Adipogenic Effect on 3T3-L1 Preadipocytes

**Figure 1:**
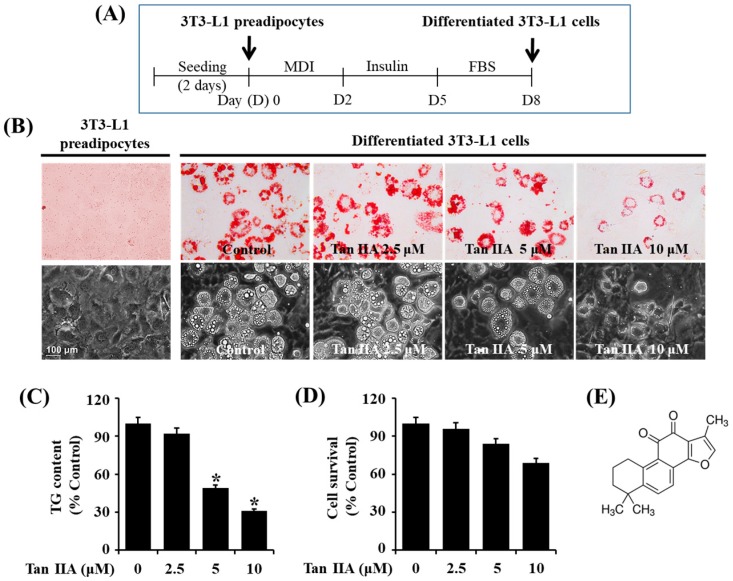
Effects of tanshinone IIA on lipid accumulation, triglyceride (TG) contents, and cell growth during 3T3-L1 preadipocyte differentiation. (**A**) Timetable for 3T3-L1 preadipocyte differentiation; (**B**) measurement of the cellular lipid (lipid droplets) in 3T3-L1 preadipocytes (undifferentiated) or differentiated adipocytes on day 8 of differentiation (D8) by Oil Red O staining. Phase-contrast images of the cells were also taken after the treatment (lower panels in B); (**C**) quantification of the cellular TG contents in tanshinone IIA-treated 3T3-L1 preadipocytes on D8 by AdipoRed assay. Values are mean ± SE of data from three independent experiments with three replicates. * *p* < 0.05 vs. control (no chemical); (**D**) 3T3-L1 preadipocytes were grown under the above-mentioned 3T3-L1 preadipocyte differentiation condition in Figure 1A. On day 8 (D8), the tanshinone IIA-treated 3T3-L1 preadipocytes, which cannot be stained with trypan blue dye, was counted under microscope. The cell count assay was done in triplicates. Data are mean ± SE of three independent experiments. * *p* < 0.05 vs. control (no chemical); (**E**) is the chemical structure of tanshinone IIA.

Initially, the treatment effect of various concentrations of tanshinone IIA on lipid accumulation during the differentiation of 3T3-L1 preadipocytes into adipocytes was determined by an Oil Red O staining. Timescale of 3T3-L1 preadipocyte differentiation is shown in Figure 1A. As shown in Figure 1B (upper panels), in induction medium, it was only on day eight of cell differentiation that many LDs were formed in 3T3-L1 preadipocytes, compared with no lipid droplet (LD) in undifferentiated cells. However, when compared with the mock-treated cells, treatment of the preadipocytes with tanshinone IIA for 8 days reduced the amounts of LDs in a dose-dependent manner. Apparently, 10 μM tanshinone IIA most strongly inhibited LD accumulation. The tanshinone IIA-induced lipid-reducing effect was also confirmed by light microscopic measurement (Figure 1B, lower panels). We further tested tanshinone IIA’s lipid-lowering effect on 3T3-L1 adipocytes by AdipoRed assay. As shown in Figure 1C, tanshinone IIA reduced cellular TG contents in a concentration-dependent manner during 3T3-L1 preadipocyte differentiation. Data of cell count assay revealed that tanshinone IIA also dose-dependently reduced the number of 3T3-L1 adipocytes (Figure 1D), but it was not significantly different. Consequently, the 10 μM concentration of tanshinone IIA with strong reductive effects on lipid accumulation and TG contents without significantly affecting the growth of 3T3-L1 adipocytes was chosen for further studies. 

### 2.2. Tanshinone IIA Decreases Protein and mRNA Expressions of C/EBP-α and PPAR-γ during 3T3-L1 Preadipocyte Differentiation

**Figure 2:**
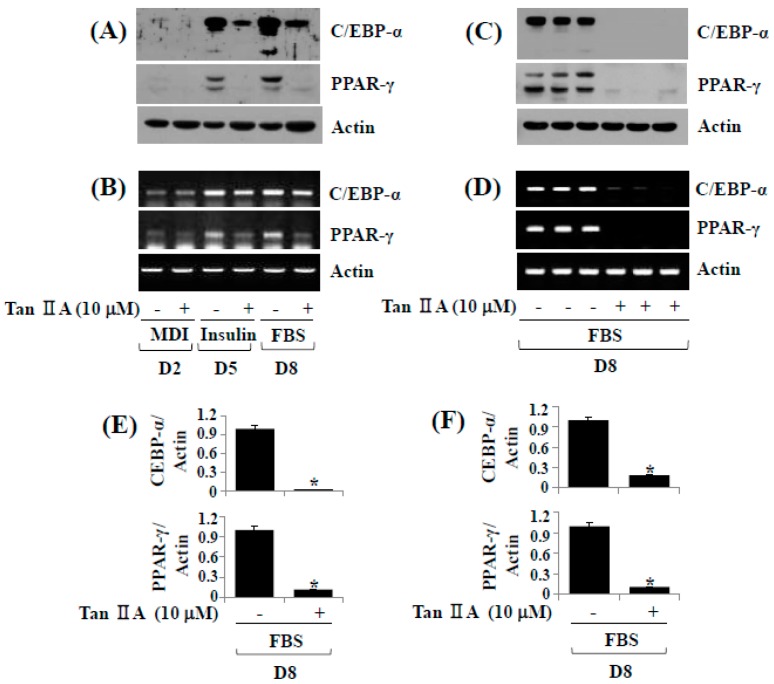
Effects of tanshinone IIA on protein and mRNA expression levels of CCAAT/enhancer-binding protein-α (C/EBP-α) and peroxisome proliferator-activated receptor-γ (PPAR-γ) during 3T3-L1 preadipocyte differentiation. (**A**,**B**) 3T3-L1 preadipocytes were differentiated with induction medium containing a cocktail of hormones (MDI) that include 0.5 mM IBMX (M) 0.5 μM dexamethasone (**D**) and 5 μg/mL insulin (I) insulin, and fetal bovine serum (FBS) in the presence or absence of tanshinone IIA, and harvested at days 2, 5 and 8, respectively. Total cellular protein and RNA at the indicated time point were extracted and analyzed by Western blot (**A**) and reverse transcription-polymerase chain reaction (RT-PCR (**B**) analysis, respectively; (**C**,**D**) Western blot (**C**) and RT-PCR (**D**) analysis in triplicate experiments on D8, respectively; (**E**,**F**) The densitometry data of (**C**,**D**), respectively. * *p* < 0.05 compared to the value of tanshinone IIA free control at the indicated day.

To understand molecular/cellular mechanisms underlying the tanshinone IIA-induced anti-adipogenic effect, we then examined its effect on protein expressions of C/EBP-α and PPAR-γ adipogenic transcription factor by Western blot analysis. Tanshinone IIA (10 μM) markedly suppressed protein expressions of C/EBP-α and PPAR-γ especially on days five and eight (Figure 2A). We next determined whether tanshinone IIA modulates mRNA expressions of C/EBP-α and PPAR-γ by reverse transcription-polymerase chain reaction (RT-PCR) analysis. Tanshinone IIA also largely inhibited mRNA expressions of C/EBP-α and PPAR-γ on days five and eight (Figure 2B). Triplicate experiments confirmed the ability of tanshinone IIA to strongly inhibit protein and mRNA expressions of C/EBP-α and PPAR-γ on day eight of differentiation (Figure 2C,D). The densitometry data of Figure 2C,D are shown in Figure 2E,F. 

### 2.3. Tanshinone IIA Reduces Phosphorylation Levels of STAT-3/5 during 3T3-L1 Preadipocyte Differentiation

**Figure 3:**
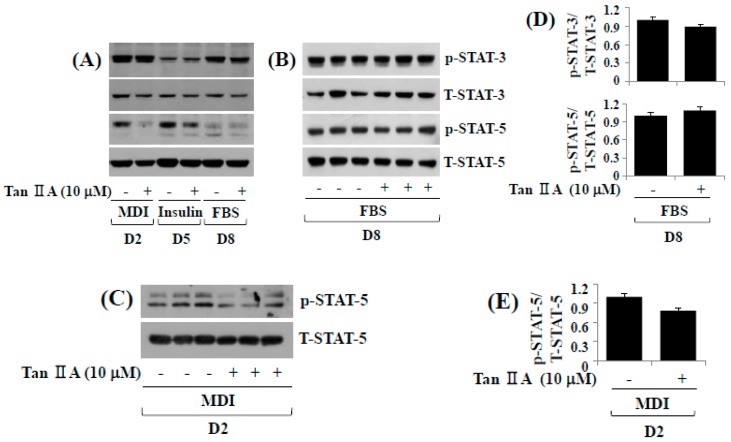
Effects of tanshinone IIA on phosphorylation and expression levels of STAT-3/5 during 3T3-L1 preadipocyte differentiation. (**A**) 3T3-L1 preadipocytes were differentiated with induction medium containing MDI, insulin and FBS in the presence or absence of tanshinone IIA, and harvested at D2, D5, and D8, respectively. Total cellular protein at the indicated time point were extracted and analyzed by Western blot analysis; (**B**) Western blot analysis in triplicate experiments on D8; (**C**) Western blot analysis in triplicate experiments on D2; (**D**,**E**) the densitometry data of (**B**,**C**), respectively.

In addition to C/EBP-α and PPAR-γ, the family of STATs including STAT-3 and STAT-5 plays crucial roles in 3T3-L1 adipocyte differentiation [15]. This promptly led us to investigate the effect of tanshinone IIA on expression and/or activity (phosphorylation) of STAT-3 and STAT-5 during 3T3-L1 preadipocyte differentiation by Western blot analysis. While tanshinone IIA did not modulate phosphorylation and total expression levels of STAT-3 protein on days two and five of adipocyte differentiation, it slightly reduced phosphorylation levels of STAT-3 without affecting the protein expression levels on day eight of adipocyte differentiation (Figure 3A). Tanshinone IIA also reduced phosphorylation levels of STAT-5 protein on days two and five, but not day eight, of adipocyte differentiation. Total protein expression of STAT-5 remained unchanged under these experimental conditions. In triplicate experiments, tanshinone IIA slightly reduced phosphorylation levels of STAT-3, but not STAT-5, on day eight of adipocyte differentiation (Figure 3B). However, we observed a more pronounced reduction of STAT-5 phosphorylation on day two of adipocyte differentiation (Figure 3C). The densitometry data of Figure 3B,C also are shown in Figure 3D,E, respectively.

### 2.4. Tanshinone IIA Reduces Protein and/or mRNA Expressions of FAS, Perilipin A, Leptin, and Resistin during 3T3-L1 Preadipocyte Differentiation

**Figure 4:**
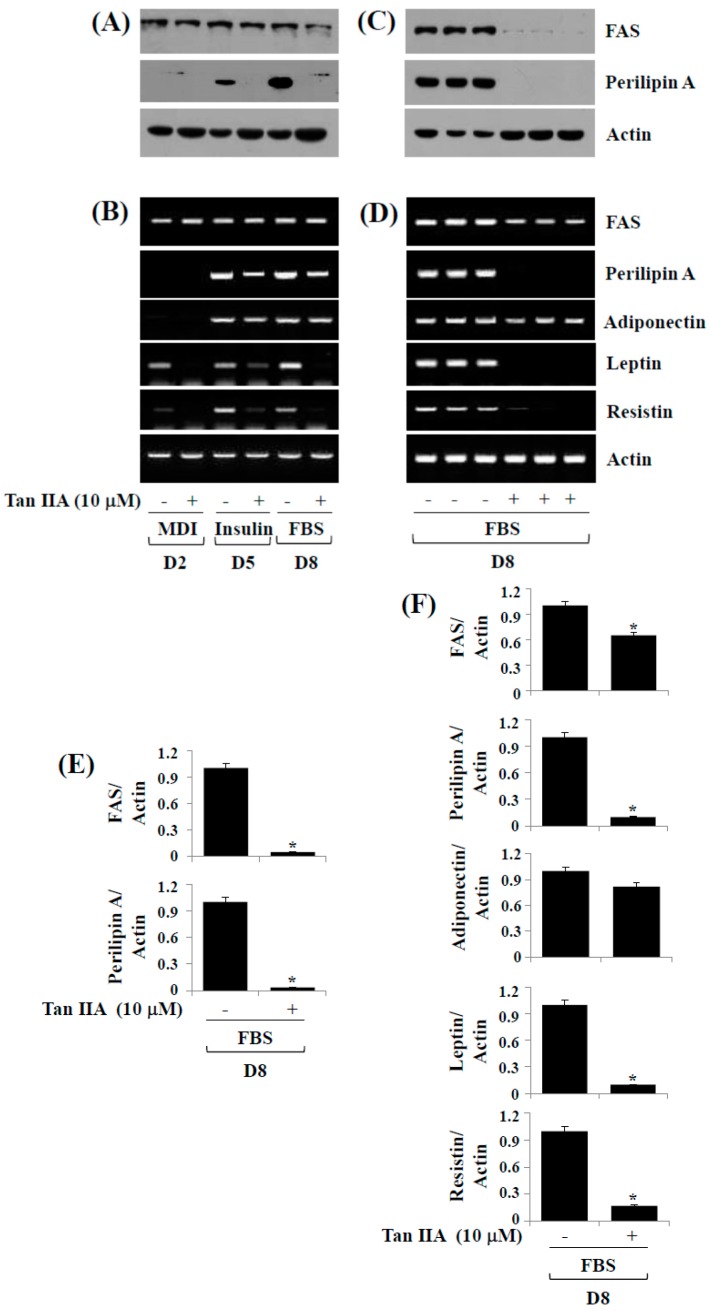
Effects of tanshinone IIA on protein and/or mRNA expression levels of FAS, perilipin A, leptin, and resistin during 3T3-L1 preadipocyte differentiation. (**A**,**B**) 3T3-L1 preadipocytes were induced to differentiate with induction medium containing MDI, insulin, and FBS in the presence or absence of tanshinone IIA, and harvested at D2, D5, and D8, respectively. Total cellular protein and mRNA at the indicated time point were extracted and analyzed by Western blot (**A**) and RT-PCR (**B**) analysis, respectively; (**C**,**D**) Western blot (**C**) and RT-PCR (**D**) analysis in triplicate experiments on D8, respectively; (**E**,**F**) The densitometry data of (**C**,**D**), respectively. * *p* < 0.05 compared to the value of tanshinone IIA free control at the indicated day.

Increased expression of adipocyte-specific genes (FAS) and LD-interacting proteins (perilipin A) occurs during 3T3-L1 preadipocyte differentiation. We next sought to explore whether tanshinone IIA alters the protein and/or mRNA expressions of FAS and perilipin A during 3T3-L1 preadipocyte differentiation. Tanshinone IIA greatly suppressed protein levels of FAS and perilipin A on days two, five and/or eight of adipocyte differentiation (Figure 4A). Tanshinone IIA also reduced mRNA levels of FAS and perilipin A on days five and/or eight of adipocyte differentiation (Figure 4B). We next measured the effect of tanshinone IIA on mRNA expressions of adipokines (leptin, adiponectin, and resistin) during 3T3-L1 preadipocyte differentiation. Notably, tanshinone IIA strongly repressed mRNA expression of leptin and resistin, but not adiponectin, on days two, five, and eight. Triplicate experiments confirmed the ability of tanshinone IIA to inhibit protein expressions of FAS and perilipin A (Figure 4C) and mRNA expressions of FAS, perilipin A, leptin, and resistin (Figure 4D) on day eight of adipocyte differentiation. The densitometry data of Figure 4C,D are shown in Figure 4E,F, respectively. 

### 2.5. Tanshinone IIA Does Not Induce Lipolysis in Differentiated 3T3-L1 Adipocytes 

**Figure 5:**
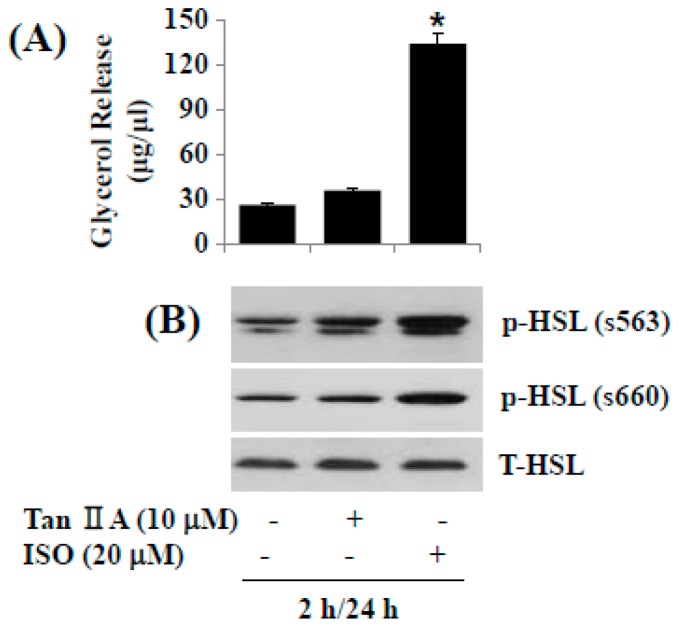
Effects of tanshinone IIA on lipolysis and hormone-sensitive lipase (HSL) phosphorylation in differentiated 3T3-L1 cells. (**A**) Differentiated 3T3-L1 cells were serum-starved for 2 h and treated with tanshinone IIA or isoproterenol (ISO) at the indicated concentration for additional 24 h. Glycerol contents were measured in triplicates. Data are mean ± SE of three independent experiments. * *p* < 0.05 vs. control; (**B**) Differentiated 3T3-L1 cells were serum-starved for 2 h and treated with tanshinone IIA or ISO at the indicated concentration for additional 24 h. Cellular protein was extracted and analyzed by Western blot analysis.

We next investigated the effect of tanshinone IIA on lipolysis in differentiated 3T3-L1 adipocytes. In this study, the tanshinone IIA’s lipolytic effect was assessed by glycerol contents in culture medium from the tanshinone IIA-treated cells. For comparison, isoproterenol (ISO), a known lipolytic agent [28], was included as a positive control. As anticipated, ISO largely stimulated glycerol release in differentiated 3T3-L1 adipocytes (Figure 5A), but tanshinone IIA did not induce it. Moreover, while ISO strongly increased lipolysis-related hormone-sensitive lipase (HSL) phosphorylation on S563 and S660 in differentiated 3T3-L1 adipocytes (Figure 5B), tanshinone IIA did not largely modulate HSL phosphorylation on S563 and S660. ISO or tanshinone IIA treatment did not affect total HSL protein level in differentiated 3T3-L1 adipocytes. 

### 2.6. Effect of Tanshinone IIA on Lipid Accumulation in Zebrafish 

**Figure 6:**
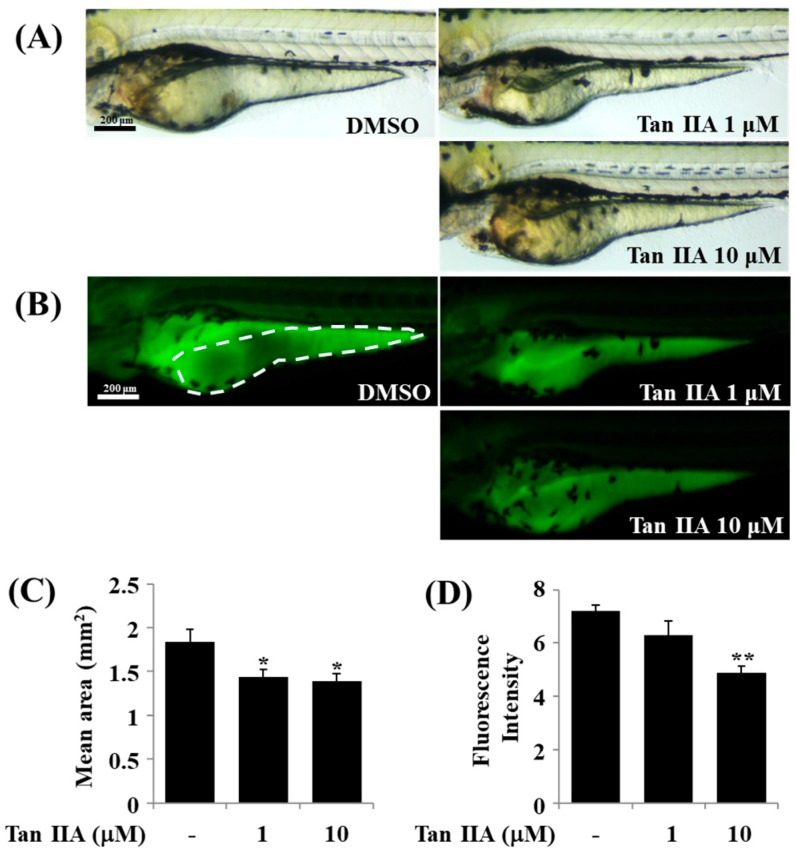
Effect of tanshinone IIA on lipid accumulation in zebrafish. Two day post fertilization (dpf) larvae were exposed in the absence (DMSO) or presence of tanshinone IIA at the indicated concentrations. After 24 h, three dpf larvae were stained with LipidGreen2 and visualized under bright field (**A**) and fluorescence field (**B**); (**C**,**D**) Size and intensity of yolk (white dash in B) were quantified using the ImageJ software, respectively. Graph bars indicate mean ± SEM (standard error of the mean). Unpaired *t*-test was used to analyze difference versus DMSO group, and statistical significance was set 0.05 and 0.01 (* *p* ≤ 0.05, ** *p* ≤ 0.01). *n* = 10.

Zebrafish is a vertebrate model that has been widely used to study lipid biology including lipid metabolism and the genes regulating lipid processing [29]. We have recently synthesized LipidGreen2 that selectively stains fat deposits in live zebrafish [30,31], which may support the relevance of use of this zebrafish-based LipidGreen2 staining in evaluating the tanshinone IIA’s lipid-reducing effect. To see its efficacy in vivo, we next investigated whether tanshinone IIA inhibits lipid accumulation in zebrafish by LipidGreen2 staining. To this end, two day post fertilization (dpf) larvae were exposed to DMSO or different concentrations of tanshinone IIA for 24 h. The conditioned larvae were washed, anesthetized, and stained with Lipid Green2 and visualized under bright field (Figure 6A) and fluorescence field (Figure 6B). Strong fluorescence was observed in control group treated with DMSO (Figure 6B). However, treatment with tanshinone IIA showed a dose-dependent reduction level of fluorescence intensity. Reduction of fluorescence was indicative of inhibition of lipid accumulation. The reduction in size and intensity of yolk after treatment with tanshinone IIA were confirmed using the Image J software (Figure 6C,D). 

## 3. Discussion

Excessive preadipocyte differentiation and the resultant high fat accumulation in the adipose tissue are closely linked to the development of obesity. Thus, inhibitors of preadipocyte differentiation may have preventive and therapeutic potential as anti-obesity drugs. In this study, we demonstrated that tanshinone IIA has strong anti-adipogenic effects on 3T3-L1 preadipocytes and zebrafish, and the anti-adipogenic effect of tanshinone IIA on 3T3-L1 cells is mediated through control of the expression and/or phosphorylation levels of C/EBP-α, PPAR-γ, FAS, perilipin A, and STAT-3/5.

Aforementioned, the in vitro and in vivo anti-obesity effects of tanshinone IIA have been previously reported [22], as evidenced by its ability to inhibit lipid accumulation in 3T3-L1 cells and to reduce body weight in a high fat diet-induced obese mouse. In this study, we also showed that tanshinone IIA at 10 μM concentration largely blocked lipid accumulation in differentiating 3T3-L1 cells, and its lipid-lowering effect was also seen in zebrafish, which further supports the notion of tanshinone IIA-mediated in vitro and in vivo anti-adipogenic effects. 

Mounting evidence suggests that the differentiation of 3T3-L1 preadipocyte into adipocytes is largely controlled by the expressions and/or activities of the family of adipogenic transcription factors, including C/EBPs, PPARs, and STATs [12,13,32,33,34,35]. Previously, it has been reported that the anti-adipogenic effect of tanshinone IIA on 3T3-L1 cells is mediated through down-regulation of C/EBP-α at the protein and mRNA levels and inhibition of PPAR-γ activity and its downstream pathway (PPAR-γ antagonism), as assessed by its ability to suppress the gene expression of PPAR-γ’s targets (aP2, CD36, lipoprotein lipase, and uncoupling protein 2) [22]. However, the present study shows that tanshinone IIA inhibited the mRNA and protein expressions of both C/EBP-α and PPAR-γ during 3T3-L1 adipocyte differentiation. These results suggest that tanshinone IIA lowers cellular levels of C/EBP-α and PPAR-γ by transcriptional repression, and the anti-adipogenic effect of tanshinone IIA on 3T3-L1 cells is closely linked to down-regulation of the expression levels of these transcription factors. The JAK-STAT signaling pathway mediates a number of physiological and pathological processes including development, hematopoiesis, and inflammation. Although the JAK-STAT signaling pathway occurs in all cells, it is known that phosphorylation levels of STAT-3/5 are markedly elevated during 3T3-L1 adipocyte differentiation [9,14,15]. In the present study, we have shown that tanshinone IIA reduces the phosphorylation of STAT-3/5 during 3T3-L1 adipocyte differentiation. Consequently, the reduced expression and phosphorylation levels of STAT-3/5 during 3T3-L1 preadipocyte differentiation should be suggested as part of the tanshinone IIA-mediated anti-adipogenic effect on 3T3-L1 cells. Furthermore, considering the role of the JAK–STAT signaling pathway in adipose tissue macrophage recruitment and development [36], further studies investigating the effect of tanshinone IIA on the recruitment of M1 macrophage are encouraged. 

Other studies have recently shown that there is a time-dependent increase in the expression of FAS and perilipin A during 3T3-L1 preadipocyte differentiation [10,37,38]. Among those, FAS is a key lipogenic enzyme that catalyzes all steps of the biosynthesis of long chain fatty acids and its overexpression is observed in cells or tissues with high rates of fatty acid synthesis [18]. Perilipin A is a highly phosphorylated protein in adipocytes that are not secreted, but localized at the surface of LDs [17]. In addition, there is strong evidence that perilipin A binds to and stabilizes the newly formed LDs during 3T3-L1 preadipocyte differentiation [14,19], and perilipin A expression in human adipose tissue is elevated with obesity [17]. Thus, inhibition of the expression and/or activity of FAS and perilipin A is a viable option for the treatment with obesity. In a previous study using an in vitro enzyme assay, it was demonstrated that tanshinone IIA (and other tanshinones) inhibits the enzyme activity of FAS with IC_50_ values ranging from 12.0 to 30.3 μM [33]. Little is known about tanshinone IIA regulation of perilipin A expression in adipocytes. In this study, however, we found that tanshinone IIA at 10 μM concentration greatly reduced the protein and mRNA levels of both FAS and perilipin A on day eight of differentiation. These results prompt us to think that tanshinone IIA reduces cellular levels of FAS and perilipin A by transcriptional down-regulation, and reduction of FAS and perilipin A expressions may further contribute to tanshinone IIA-mediated anti-lipogenic and anti-adipogenic effects on 3T3-L1 cells.

Adipose tissue is a major source of adipokines including adiponectin, leptin, and resistin [39,40]. Reportedly, while adiponectin expression levels decrease with increase in the adiposity [40,41], lepin and resistin levels increase in obesity [40,42,43]. The adipocytes-derived hormone resistin is postulated to be linked to obesity, insulin resistance and diabetes [44]. Consequently, suppression of leptin expression is an alternative against obesity. In this study, we showed that tanshinone IIA strongly reduced cellular transcripts of leptin and resistin, but not adiponectin, during 3T3-L1 preadipocyte differentiation. These results may suggest that tanshinone IIA could be a useful lead for future drug development efforts for the treatment of obesity and related disorders associated with the overexpression of leptin and resistin.

Lipolysis is defined as the hydrolytic cleavage of ester bonds in TG, resulting in the generation of fatty acids and glycerol [45]. HSL is a key enzyme in the mobilization of fatty acids from stored TG [16]. Accordingly, lipolysis is induced by activation of protein kinase A which phosphorylates HSL on S563, S659, and S660 in adipocytes [46]. However, considering the present findings that tanshinone IIA did not induce glycerol release and HSL phosphorylation (S563, S660) in differentiated 3T3-L1 cells, it is evident that tanshinone IIA has no lipolytic effect on differentiated 3T3-L1 cells. 

In sum, we demonstrated that tanshinone IIA has strong anti-adipogenic effects on 3T3-L1 preadipocytes and zebrafish. The anti-adipogenic effect of tanshinone IIA on 3T3-L1 cells is mediated through down-regulation of the expression and/or phosphorylation levels of C/EBP-α, PPAR-γ, FAS, perilipin A, and STAT-3/5. The present findings advocate this natural phytochemical tanshinone IIA as a potential therapeutic path against obesity.

## 4. Materials and Methods 

### 4.1. Materials

Polyclonal C/EBP-α antibody, monoclonal PPAR-γ antibody, monoclonal STAT-3 antibody, monoclonal phospho-STAT-3 (p-STAT-3) antibody, polyclonal STAT-5 antibody, and polyclonal phospho-STAT-5 (p-STAT-5) antibody were purchased from Santa Cruz Biotechnology (Santa Cruz, CA, USA). Polyclonal p-HSL (S563), polyclonal p-HSL (S565) and polyclonal p-HSL (S660) were purchased from Cell Signaling Technology (Danvers, MA, USA). Polyclonal HSL antibody was purchased from Cayman chemical. (Ann Arbor, MI, USA). Monoclonal FAS antibody was purchased from BD Bioscience (San Jose, CA, USA). The active compound Tanshinone IIA was purchased from Selleckchem (Houston, TX, USA). Monoclonal β-actin antibody was purchased from Sigma (St. Louis, MO, USA). Polyclonal perilipin A antibody was purchased from Bio Vision Inc. (Milpitas, CA, USA). 

### 4.2. 3T3-L1 Cell Culture and Differentiation

3T3-L1 murine white preadipocytes (ATCC, Manassas, VA, USA) were grown up to the contact inhibition stage and remained in the post-confluent stage for 2 days in Dulbecco Modified Eagle Medium (DMEM) supplemented with 10% calf bovine serum (Gibco, Gaithersburg, MD, USA) and penicillin-streptomycin (Welgene, Daegu, Korea). Differentiation was then induced by changing the medium to DMEM supplemented with 10% FBS (Welgene) plus a cocktail of hormones (MDI) that include 0.5 mM IBMX (M) (Sigma, St. Louis, MO, USA), 0.5 μM dexamethasone (D) (Sigma) and 5 μg/mL insulin (I) (Sigma) in the presence or absence of tanshinone IIA at the indicated concentrations. After 48 h MDI-induction, the differentiation medium was replaced with DMEM supplemented with 10% FBS and 5 μg/mL insulin in the presence or absence of tanshinone IIA at the indicated concentrations. The cells were then fed every other day with DMEM containing 10% FBS in the presence or absence of tanshinone IIA at the indicated concentrations until day eight. On day eight, the preadipocytes became mature adipocytes that rounded-up and filled with many oil droplets.

### 4.3. Oil Red O Staining

On day eight of differentiation, control or tanshinone IIA-treated 3T3-L1 cells were washed twice with PBS, fixed with 10% formaldehyde for 2 h at room temperature, washed with 60% isopropanol and dried completely. The fixed cells were then stained with Oil red O working solution for 1 h at room temperature (RT) and then washed twice with distilled water. Lipid droplets were observed by light microscopy (Nikon, Tokyo, Japan).

### 4.4. Cell Count Analysis

3T3-L1 preadipocytes that were seeded in 24-well plates were similarly grown under the above-mentioned differentiation conditions. On day eight of differentiation, control or tanshinone IIA-treated 3T3-L1 cells, which cannot be stained with trypan blue dye, was counted under microscope. The cell count assay was done in triplicates. Data are mean ± standard error (SE) of three independent experiments.

### 4.5. Quantification of Intracellular TG Content by Adipored Assay

On day eight of differentiation, lipid content in control or tanshinone IIA-treated 3T3-L1 cells was measured using a commercially available AdipoRed Assay Reagent kit according to the manufacturer’s instructions (Lonza, Basel, Switzerland). After a 10 min incubation, fluorescence was measured on Victor^3^ (Perkin Elmer, Waltham, MA, USA) with an excitation at 485 nm and an emission at 572 nm.

### 4.6. Preparation of Whole Cell Lysates

At the designated time point, 3T3-L1 cells were washed twice with PBS and exposed to a modified RIPA buffer (50 mM Tris-Cl (pH 7.4), 150 mM NaCl, 0.1% sodium dodecyl sulfate, 0.25% sodium deoxycholate, 1% Triton X-100, 1% Nonidet P-40, 1 mM EDTA, 1 mM EGTA, proteinase inhibitor cocktail (1×)). The cell lysates were collected and centrifuged at 12,000 rpm for 20 min at 4 °C. The supernatant was saved and protein concentrations were determined with Bradford reagent (Bio-Rad, Hercules, CA, USA).

### 4.7. Western Blot Analysis

Proteins (50 μg) were separated by SDS-PAGE (10%) and transferred onto nitrocellulose membranes (Millipore, Billerica, MA, USA). The membranes were washed with TBS (10 mM Tris, 150 mM NaCl) supplemented with 0.05% (*v*/*v*) Tween 20 (TBST) followed by blocking with TBST containing 5% (*w*/*v*) non-fat dried milk. The membranes were incubated overnight with antibodies specific for C/EBP-α (1:1000), PPAR-γ (1:1000), STAT-3 (1:2000), p-STAT-3 (1:2000), STAT-5 (1:2000), p-STAT-5 (1:2000), FAS (1:1000), perilipin A (1:2000), p-HSL (S563, S565, S660, 1:1000), HSL (1:1000), or β-actin (1:10,000) at 4 °C. The membranes were then exposed to secondary antibodies coupled to horseradish peroxidase for 2 h at RT. The membranes were washed three times with TBST at RT. Immunoreactivities were detected by ECL reagents. Equal protein loading was assessed by the expression level of actin protein. 

### 4.8. Reverse Transcription-Polymerase Chain Reaction (RT-PCR)

At the designated time point, total cellular RNA in control or tanshinone IIA-treated 3T3-L1 cells was isolated with the RNAzol-B (Tel-Test, Friendswood, TX, USA). Three micrograms of total RNA were reverse transcribed using a random hexadeoxynucleotide primer and reverse transcriptase. Single stranded cDNA was amplified by PCR with the following primers. Primer sequences used for amplifications were as follows: C/EBP-α sense 5′-TTACAACAGGCCAGGTTTCC-3′; C/EBP-α antisense 5′-CTCTGGGATGGATCGATTGT-3′; PPAR-γ sense 5′-GGTGAAACTCTGGGAGATTC-3′; PPAR-γ antisense 5′-CAACCATTGGGTCAGCTCTC-3′; FAS sense 5′-TTGCTGGCACTACAGAATGC-3′; FAS antisense 5′-AACAGCCTCAGAGCGACAAT-3′; perilipin A sense 5′-CTTTCTCGACACACCATGGAAACC-3′; perilipin A antisense 5′-CCACGTTATCCGTAACACCCTTCA-3′; Adiponectin sense 5′-GGAGATGCAGGTCTTCTTGGT-3′; Adiponectin antisense 5′-TCCTGATACTGGTCGTAGGTGAA-3′; Leptin sense 5′-CCAAAACCCTCATCAAGACC-3′; Leptin antisense 5′-CTCAAAGCCACCACCTCTGT-3′; Resistin sense 5′-CCGATGAGCAGTCACCTCCA-3′; Resistin antisense 5′-CAGCTGCTTCGCCTCGTCCTCCT-3′; Actin sense 5′-TCATGAAGTGTGACGTTGACATCCGT-3′; Actin antisense 5′-CCTAGAAGCATTTGCGGTGCACGATG-3′. The PCR conditions applied were: C/EBP-α, 30 cycles of denaturation at 95 °C for 30 s, annealing at 62 °C for 30 s, and extension at 72 °C for 30 s; PPAR-γ, 30 cycles of denaturation at 95 °C for 30 s, annealing at 53 °C for 30 s, and extension at 72 °C for 30 s; FAS, 30 cycles of denaturation at 95 °C for 15 s, annealing at 55 °C for 40 s, and extension at 68 °C for 45 s; perilipin A, 30 cycles of denaturation at 95 °C for 1 min, annealing at 55 °C for 1 min, and extension at 72 °C for 1 min; Adiponectin, 30 cycles of denaturation at 95 °C for 1 min, annealing at 53.5 °C for 1 min, and extension at 72 °C for 1 min; leptin, 30 cycles of denaturation at 95 °C for 1 min, annealing at 57 °C for 1 min, and extension at 72 °C for 1 min; Resistin, 35 cycles of denaturation at 95 °C for 1 min, annealing at 57 °C for 1 min, and extension at 72 °C for 1 min; Actin, 25 cycles of denaturation at 95 °C for 30 s, annealing at 57 °C for 30 s, and extension at 72 °C for 1 min. Expression levels of actin mRNA were used as an internal control to evaluate the relative mRNA expression of adipocyte-specific genes and adipokines. 

### 4.9. Measurement of Glycerol Contents

Differentiated 3T3-L1 adipocytes were serum-starved for 2 h and treated with tanshinone IIA or isoproterenol (ISO) for 24 h. Culture medium was saved and subjected to measure glycerol contents with a free glycerol reagent (Sigma) according to the manufacturer’s instructions. The optical absorbance was determined at wavelength of 540 nm using the microplate reader. 

### 4.10. Zebrafish Experiment

Zebrafish was maintained under standard conditions as previously described [47]. All experimental protocols involving the zebrafish were approved by the Animal Care and Committee of the Korea Research Institute of Chemical Technology. Two dpf (day post fertilization) larvae were arrayed in 24-well plate in which each well contains 10 larvae (*n* = 10) and 1 mL egg water (0.6 g/L sea salt). The indicated concentrations of tanshinone IIA dissolved in dimethyl sulfoxide (DMSO) were added to each well and incubated in 37 °C incubator. After 24 h, larvae were washed out with egg water and stained with 5 μM LipidGreen2 solution [30]. After 10 min, larvae were washed out three times with egg water. To acquire images, larvae were anesthetized in tricaine (Sigma) and mounted in 3% methylcellulose (Sigma). Images were acquired under Leica MZ10 F stereomicroscope, Leica DFC425 camera, and Leica Application Suite software v4.5 (Leica Microsystems, Wetzlar, Germany). Yolk size and fluorescence intensity were measured by ImageJ software [48]. Data are mean ± SEM (standard error of the mean). 

### 4.11. Statistical Analyses

Cell count analysis was done in triplicates and repeated three times. Data were expressed as means ± SE (standard error). The significance of difference was determined by One-Way ANOVA. All significance testing was based upon a *p* value of <0.05. For measurement of the size and intensity of yolk in zebrafish, unpaired *t*-test was used to analyze difference versus DMSO group (*n* = 10). The statistical significance was based upon a *p* value of ≤0.05 or ≤0.01. 

## Abbreviations

C/EBP-α	CCAAT/enhancer-binding protein-α
ECL	enhanced chemiluminescence
FAS	fatty acid synthase
FBS	fetal bovine serum
IBMX	3-isobutyl-1-methylxanthine
LD	lipid droplet
HSL	Hormone-sensitive lipase
PPAR-γ	peroxisome proliferator-activated receptor-γ
TG	triglyceride
STAT	signal transducer and activator of transcription
RT-PCR	Reverse transcription-polymerase chain reaction
RT	Room Temperature
